# Meta-Analysis of the Incidence, Prevalence, and Correlates of Atrial Fibrillation in Rheumatic Heart Disease

**DOI:** 10.5334/gh.807

**Published:** 2020-05-18

**Authors:** Jean Jacques Noubiap, Ulrich Flore Nyaga, Aude Laetitia Ndoadoumgue, Jan René Nkeck, Anderson Ngouo, Jean Joel Bigna

**Affiliations:** 1 Centre for Heart Rhythm Disorders, South Australian Health and Medical Research Institute (SAHMRI), University of Adelaide and Royal Adelaide Hospital, Adelaide, AU; 2 Department of Internal Medicine and Specialties, Faculty of Medicine and Biomedical Sciences, Yaoundé, CM; 3 School of Health and Related Research, The University of Sheffield, Sheffield, UK; 4 Department of Epidemiology and Public Health, Centre Pasteur of Cameroon, Yaoundé, CM; 5 Faculty of Medicine, University of Paris Sud XI, Le Kremlin-Bicêtre, FR

**Keywords:** atrial fibrillation, rheumatic heart disease, prevalence, incidence, risk factors

## Abstract

**Objective:**

To estimate the incidence, prevalence, and correlates of atrial fibrillation (AF) in a global population with rheumatic heart disease (RHD).

**Methods:**

Bibliographic databases were searched to identify all published studies providing data on AF in patients with RHD. Random-effects meta-analysis method was used to pool estimates.

**Results:**

Eighty-three studies were included, reporting data from 75,637 participants with RHD in 42 countries. The global prevalence of AF in RHD was 32.8% (range: 4.3%–79.9%). It was higher in severe valvular disease (30.8% vs 20.7%, p = 0.009), in severe mitral valve disease compared to severe aortic disease (30.4% vs 6.3%, p = 0.038). The global cumulative incidence of AF in patients with RHD was 4.8%, 11.4%, 13.2%, and 30.8% at 1, 2, 5, and 10 years of follow-up, respectively. From comparison between patients with and without AF, AF was associated with increased age (mean difference [MD]: 9.5 years; 95% CI: 7.8–1.3), advanced heart failure (odds ratio [OR]: 4.4; 95% CI 2.1–9.3), tricuspid valve involvement (OR: 4.0; 95% CI: 3.0–5.3), history of thromboembolism (OR: 6.2; 95% CI: 3.4–11.4), highly sensitive C-reactive protein (MD: 5.5 mg/dL; 95% CI: 1.2–9.8), systolic pulmonary arterial pressure (MD: 3.6 mmHg; 95% CI: 0.8–6.3), right atrium pressure (MD: 1.5 mmHg; 95% CI: 1.0–2.0), and left atrium diameter (MD: 8.1 mm; 95% CI: 5.5–10.7).

**Conclusions:**

About one-third of patients with RHD have AF, with an incidence which almost triples every five years after diagnosis. Factors associated with AF include age, advanced heart failure, thromboembolism, and few cardiac hemodynamics parameters.

## Introduction

Rheumatic heart disease (RHD) is a leading global health problem, which disproportionally affects low- and middle-income countries (LMICs) [1]. There are nearly 33 million people living with RHD, with about 80% of them residing in LMICs [2]. RHD accounts for about 275,000 deaths every year, 95% of these occurring in LMICs [2]. RHD results from recurrent episodes of acute rheumatic fever, an autoimmune response to untreated group A streptococcal pharyngitis that causes inflammation and fibrosis of the heart valves. Severe valvular damage leads to altered hemodynamics, chamber remodeling, subsequently heart failure, pulmonary hypertension, atrial fibrillation (AF), thromboembolism, infective endocarditis, and eventually premature death [1].

AF, which is the most sustained cardiac arrhythmia in the general population, is a major problem in RHD, owing to its prevalence and complications [1]. AF is a factor of progression and decompensation of heart failure in RHD, and is associated with cardioembolic stroke and systemic emboli [1,3,4,5]. Furthermore, AF has been identified as a predictor of event-free survival before and after mitral valve intervention [4,5,6,7]. Several clinical, echocardiographic, and hemodynamic factors have been associated with the occurrence of AF in patients with RHD. Those factors include age, cardiac chambers sizes and pressures, left ventricular function, mitral valve involvement, and inflammatory markers such as highly sensitive C-reactive protein, amongst others [8,9,10]. However, reports on the correlation of those factors with AF in RHD have shown some inconsistencies.

The current review summarizes the available data on the incidence and prevalence of AF in the global RHD population, according to region, RHD endemicity, age group, valvular involvement and severity, and pre- and post-valvular intervention. A meta-analysis of the potential correlates of AF in RHD is also presented. To the best of our knowledge, this is the first systematic review and meta-analysis of the epidemiology of AF in RHD.

## Methods

This review was conducted as recommended in the Joanna Briggs Institute reviewer’s manual [11] and is reported in accordance with the Meta-analyses Of Observational Studies in Epidemiology guidelines [12].

### Literature search

PubMed/MEDLINE, Excerpta Medica Database (EMBASE), Web of Science, and Global Index Medicus were searched to identify all studies reporting primary data of the prevalence, incidence, or correlates of AF in people with RHD, published until April 30, 2019, irrespective of the language. The search strategy was built according to the PRESS guidelines [13], based on the combination of relevant terms including ‘rheumatic heart disease’, ‘atrial fibrillation’, and their bibliographic synonyms (Supplementary Table 1, Appendix). The reference lists of all eligible articles and relevant reviews were scrutinized to identify potential additional data sources.

**Figure 1:**
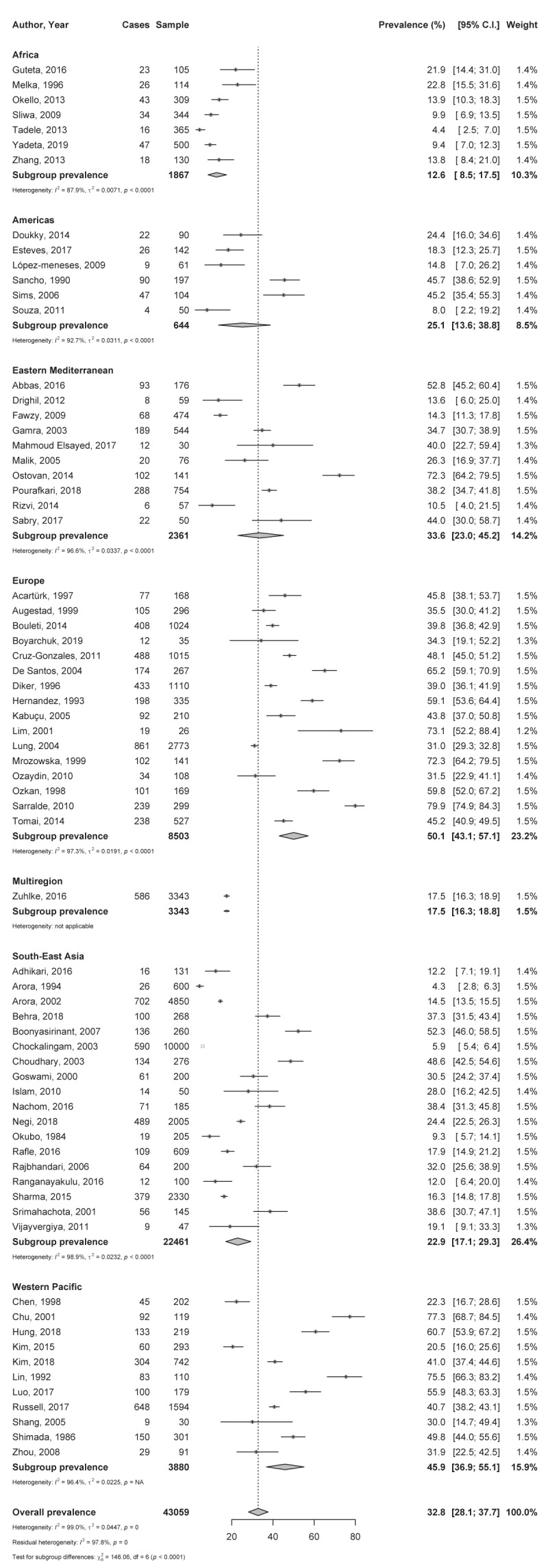
Meta-analysis of the prevalence of atrial fibrillation in rheumatic heart disease by WHO region.

### Selection of studies to include in the review

We included observational studies either cohort, case-control, or cross-sectional, reporting on the prevalence or the incidence or the correlates of AF in individuals with RHD, or enough data to compute these estimates. We excluded letters, editorials, reviews, and studies without primary data or a clear description of methods. For studies published in more than one report (duplicates), the most comprehensive reporting the largest sample size was considered.

Two investigators (JJN and JJB) independently screened records for eligibility based on titles and abstracts. Full-texts of articles deemed potentially eligible were retrieved. Further, these investigators independently assessed the full-text of each study for eligibility, and consensually retained studies to be included. Disagreements were solved through a discussion.

### Data extraction and management

Data were extracted using a preconceived, piloted, and standardized data abstraction form. Three pairs of investigators (UFN, ALN, AN, JRN, JJN, JJB) independently extracted data including: name of the first author, year of publication, study design, period of inclusion of participants, recruitment site (country, number of sites), sampling method, number of participants included with RHD, number of cases of AF, age distribution, proportion of males, and clinical presentation. We assigned a World Health Organization (WHO) geographic region and a development index to each study regarding the country of recruitment. Data were collected to compute subgroup analysis according to age group (children, adolescents, adults), valve involved (aortic and mitral), type of valvular lesion (regurgitation, stenosis, or mixed), severity of valvular lesion (mild/moderate vs severe), whether the participants have had a valvular intervention (valvulotomy, valvuloplasty, surgical valve replacement) or not, on clinical, biological, echocardiographic, and hemodynamic parameters evaluated as potential correlates of AF in RHD, including history of cerebrovascular event, heart failure, highly sensitive C reactive protein, brain natriuretic peptide, pulmonary artery pressures, right and left atrium pressures and diameters, left ventricular ejection fraction and diameters, and valvular areas.

We used an adapted version of the tool developed by the Joanna Briggs Institute to assess the risk of bias in included studies [14]. Two pairs of investigators (UFN, ALN, AN, JRN) independently ran the assessment. Discrepancies were discussed and resolved through consensus. Inter-rater agreements between investigators for study inclusion and methodological quality assessment were assessed using Cohen’s κ.

### Statistical analysis

Meta-analyses were conducted using the *meta* packages of the R statistical software (version 3.6.0, The R Foundation for statistical computing, Vienna, Austria). With *metaprop* function, we used the reference method to synthetize prevalence data as recommended by Barendregt and colleagues [15]. All prevalence estimates were reported with their 95% confidence intervals (95% CI). To minimize the effect of studies with extremely small or extremely large prevalence estimates on the overall estimate, the variance of study-specific prevalence was stabilized with the Freeman-Tukey double arcsine transformation before pooling the data with the random-effects meta-analysis model [15]. Heterogeneity was assessed by the χ^2^ test on Cochrane’s Q statistic [16], which was quantified by I^2^ values, assuming I^2^ values of 25, 50, and 75% respectively representing low, medium, and high heterogeneity [17]. The Egger test was used to assess the presence of publication bias [18]. A *p* value < 0.10 was considered indicative of a statistically significant publication bias [19]. We conducted subgroup analyses according to WHO regional location of included participants, level of human development index, valvular surgical intervention status (before and after), and age group (children, adolescents, and adults). We calculated R^2^ through meta-regression analysis (with *metareg* function) to identify covariates that explained the heterogeneity in the global estimate, and therefore quantify the heterogeneity accounted for. We conducted a leave-one-out sensitivity analysis to explore how sensitive the global prevalence of AF in RHD was to the exclusion of individual studies. We also conducted a sensitivity analysis selection only in studies with low risk of bias in the methodological assessment.

For investigating factors associated with AF, a meta-analysis using the random-effects method of DerSimonian and Laird was performed to pool weighted odds ratios (OR) and weighted mean differences (MD) with *metabin* and *metacont* functions respectively [20]. All strengths of association were reported with their 95% CI. We also performed a narrative synthesis of factors associated AF.

## Results

### Study selection and characteristics

In total, we identified 3,212 records among which 83 studies were finally included (Supplementary Figure 1) [8,9,10,21,22,23,24,25,26,27,28,29,30,31,32,33,34,35,36,37,38,39,40,41,42,43,44,45,46,47,48,49,50,51,52,53,54,55,56,57,58,59,60,61,62,63,64,65,66,67,68,69,70,71,72,73,74,75,76,77,78,79,80,81,82,83,84,85,86,87,88,89,90,91,92,93,94,95,96,97,98,99,100,101]. Agreement between review authors for study selection (κ = 0.78), and key data extraction (κ = 0.91), and methodological quality assessment (κ = 0.83) were moderate to high. Studies’ characteristics are summarized in Supplementary Table 2. For the 73 studies included in prevalence and incidence analysis, 19 (26%) studies had high risk, 30 (41%) moderate risk, and 24 (33%) low risk of bias. For the 18 studies included in the meta-analysis of correlates, one (6%) had high risk, six (33%) moderate risk, and 11 (61%) had low risk of bias. In total, data were from 75,637 participants in 42 countries. Forty-three (60%) studies collected data among patients without valvular surgical intervention, 26 (36%) among patients after valvular surgical intervention, and three (4%) studies included both patients. Data were published between 1984 and 2019. Participants were included in original studies from 1965 to 2017. The proportion of males varied between 0 and 63%. Most studies were cross-sectional (51%) and prospectively collected data (64%). Individual characteristics of included studies are in Supplementary Table 3.

**Figure 2:**
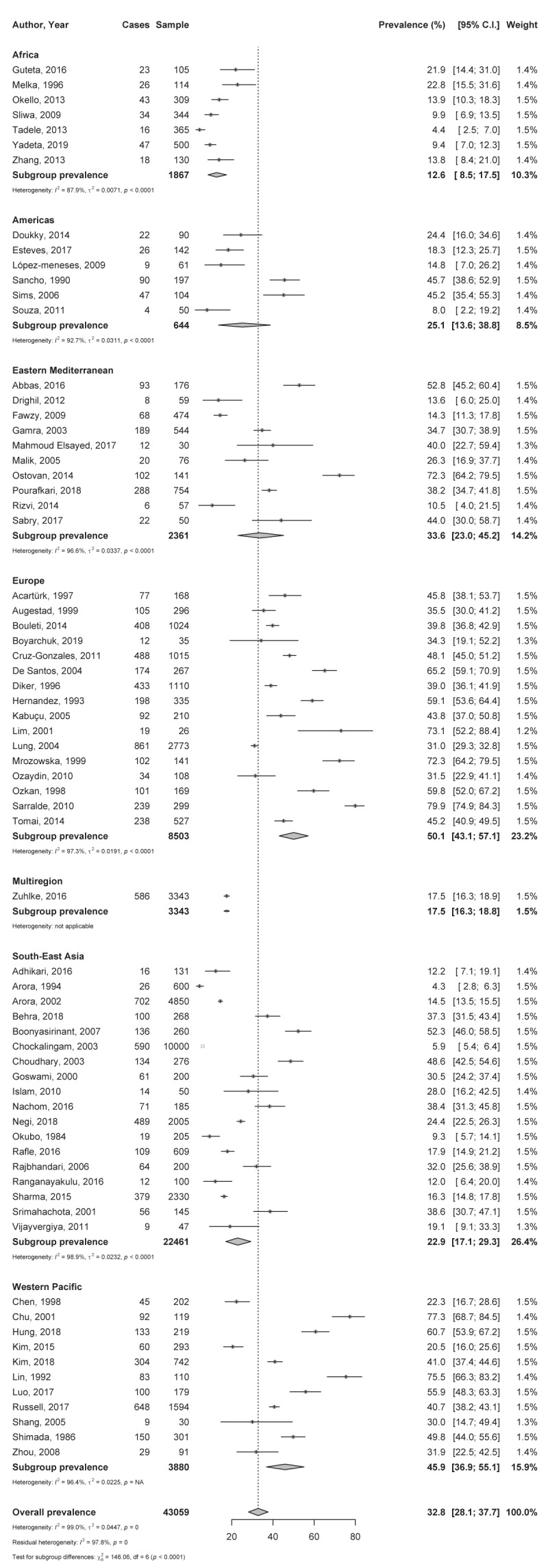
Meta-analysis of the incidence of atrial fibrillation in rheumatic heart disease.

### Global incidence and prevalence of AF in patients with RHD

The global prevalence of AF in RHD was 32.8% (95% CI 28.1–37.7) with substantial heterogeneity (Figure 1), ranging from 4.3% [54] to 79.9% [51] in individual studies. This heterogeneity was significantly explained by the country’s level of development (R^2^: 57.6%), distribution across WHO regions (R^2^: 50.4%), age groups (R^2^: 35.0%), RHD endemic profile of the country (R^2^: 24.5%), and slightly by study period (R^2^: 7.0%) and by valvular intervention status (R^2^: 3.9%) (Table 1). In a multivariate meta-regression model with all these covariates, the R^2^ was 57.1%. In the leave-one-out sensitivity analysis, all estimated global prevalences were in the range of the crude analysis (Supplementary Figure 2). When considering only low-risk bias studies, the global prevalence was very close to that of crude analysis (Table 1). The funnel plot (Supplementary Figure 3) suggested the presence of publication bias which was confirmed by the Egger test, p < 0.0001 (Table 1).

**Table 1:** Meta-analysis of the prevalence of atrial fibrillation in the global population with rheumatic heart disease.

	Prevalence, %	95% Confidence interval	N Studies	N Participants	Heterogeneity		P Egger test	P value comparison	Meta-regression	
	
					I^2^, %	P value			R^2^, %	P value for testing of moderators

**Global**	32.8	28.1–37.7	69	43,059	99.0	<0.0001	<0.0001	–	–	
· Low risk of bias studies	34.7	25.3–44.8	20	4,819	98.0	< 0.0001	0.257	–	–	
**By WHO region**									50.4	<0.0001
· Europe	50.1	43.1–57.1	16	8,503	97.3	<0.0001	0.010	<0.0001		
· Western Pacific	45.9	36.9–55.1	11	3,880	96.4	<0.0001	0.467			
· Eastern Mediterranean	33.6	23.0–45.2	10	2,361	96.6	<0.0001	0.863			
· Americas	25.1	13.6–38.8	6	644	92.7	<0.0001	0.127			
· South-East Asia	22.9	17.1–29.3	18	22,461	98.9	<0.0001	0.001			
· Africa	12.6	8.5–17.5	7	1,867	87.9	<0.0001	0.703			
**By level of human development index**									57.6	<0.0001
· Very high	45.4	38.7–52.3	18	10,498	97.8	<0.0001	0.120	<0.0001		
· High	41.9	35.5–48.3	22	5,032	95.0	<0.0001	0.760			
· Medium	21.1	16.6–26.0	23	26,006	98.5	<0.0001	0.015			
· Low	13.2	8.2–19.4	6	1,523	89.8	<0.0001	0.092			
**By endemic status**									24.5	0.0001
· Endemic area	22.8	18.5–27.3	33	28,605	98.5	<0.0001	0.002	<0.0001		
· Non-endemic area	42.8	38.1–47.6	36	14,454	96.8	<0.0001	0.205			
**By surgery valvular intervention status**									3.9	0.025
· Before valvular intervention	28.4	22.7–34.4	40	27,690	99.0	<0.0001	0.0005	0.026		
· After valvular intervention	39.3	31.8–47.0	29	15,369	98.8	<0.0001	0.008			
***Type of valvular interventions***										
- Valve replacement	60.3	48.6–71.5	4	603	87.0	<0.0001	0.701	0.0001		
- Valvuloplasty	39.5	30.6–48.8	10	3,122	95.7	<0.0001	0.503			
- Valvulotomy	28.2	19.8–37.3	11	9,467	98.5	<0.0001	0.107			
**By age group**									35.0	<0.0001
· Children and adolescents	7.6	1.9–16.8	2	496	87.8	0.004	NA	<0.0001		
· Children, adolescents, and adults	20.5	14.2–27.5	15	23,609	99.2	<0.0001	0.073			
· Adolescents and adults	26.2	17.3–36.3	6	1,774	94.2	<0.0001	0.027			
· Adults	39.7	34.4–45.1	46	17,180	98.0	<0.0001	0.021			
**By study period**									7.0	0.026
· Before 2000	38.2	30.2–46.5	35	27,825	99.4	<0.0001	< 0.0001	0.022		
· 2000 or after	27.3	22.7–32.2	34	15,234	97.4	<0.0001	0.196			

R^2^: amount of heterogeneity accounted for; NA: not applicable.

According to WHO regions, the prevalence of AF in patients with RHD varied from 12.6% (95% CI 8.5–17.5) in South-East Asia to 50.1% (43.1–57.1) in Europe, with significant difference between regions (p < 0.0001) (Figure 1 and Table 1). This prevalence increased with the country’s level of development: from 13.2% (8.2–19.4) in less developed countries to 45.4% (38.7–52.3) in the most developed countries; p < 0.0001 (Table 1). The prevalence of AF in RHD was lower in RHD endemic areas (22.8%; 18.5–27.3) compared to non-endemic (42.8%; 38.1–47.6); p < 0.0001 (Table 1). The prevalence increased with age, varying from 7.6% (1.9–16.8) in children and adolescents to 39.7% (34.4–45.1) in adults; p < 0.0001 (Table 1). Studies performed before 2000 (38.2%, 30.2–46.5) reported higher prevalence compared to studies performed in 2000 or after (27.3%, 22.7–32.2) (Table 1).

The global cumulative incidence of AF in patients with RHD was 4.8%, 11.4%, 13.2%, and 30.8% at 1, 2, 5, and 10 years of follow-up, respectively (Figure 2).

### Prevalence of AF in patients with RHD according to the clinical presentation

Patients with severe valvular disease (30.8%, 95% CI: 24.0–38.2) had higher prevalence of AF compared to those with mild/moderate disease (20.7%; 17.1–24.6), p = 0.009. The prevalence of AF was higher among patients with severe mitral valve disease (30.4%, 23.6–37.6) compared to those with severe aortic valve disease (6.3%; 0.0–26.9), p = 0.038. AF was more frequent in patients with mixed mitral valve disease (65.6%; 42.5–85.4) compared to those with mitral stenosis (33.9%; 28.5–39.4) or mitral regurgitation (21.6%; 7.8–39.7), p = 0.011. The prevalence of AF was not significantly different between patients with various forms of aortic valve disease (Table 2). The prevalence of AF in patients who had a valvular intervention (39.3%; 31.8–47.0) was higher compared to patients who did not (28.4%; 22.7–34.4); p = 0.026 (Table 1). Among patients who had a valvular intervention, those who had a surgical valve replacement (60.3%, 48.6–71.5) had higher prevalence of AF compared to those who had a valvuloplasty (39.5%, 30.6–48.8) or a valvulotomy (28.2%, 19.8–37.3); p = 0.0001 (Supplementary Figure 4).

**Table 2:** Meta-analysis of the prevalence of atrial fibrillation in the global population with rheumatic heart disease according to clinical presentation.

	Prevalence, %	95% Confidence interval	N Studies	N Participants	Heterogeneity		P Egger test	P value comparison

					I^2^, %	P value		

**Severity overall**								
· Severe valvular disease	30.8	24.0–38.2	32	14,049	98.6	<0.0001	0.027	0.009
· Moderate/mild valvular disease	20.7	17.1–24.6	1	454	NA	NA	NA	
**Severe valvular disease by valve**								
· Severe mitral disease	30.4	23.6–37.6	18	3,627	94.7	<0.0001	0.165	0.038
· Severe aortic disease	6.3	0.0–26.9	2	39	60.5	0.112	NA	
**Mitral valve disease**								
· Mixed mitral disease	65.6	42.5–85.4	4	449	94.7	<0.0001	0.461	0.011
· Mitral stenosis	33.9	28.5–39.4	42	17,221	98.1	<0.0001	0.005	
· Mitral regurgitation	21.6	7.8–39.7	6	880	96.6	<0.0001	0.528	
**Aortic valve disease**								
· Mixed aortic disease	3.6	0.8–8.1	1	110	NA	NA	NA	0.694
· Aortic stenosis	5.0	0.0–20.2	1	20	NA	NA	NA	
· Aortic regurgitation	5.8	0.6–14.4	3	180	58.3	0.091	0.398	

NA: not applicable.

### Correlates of AF in patients with RHD

**Figure 3:**
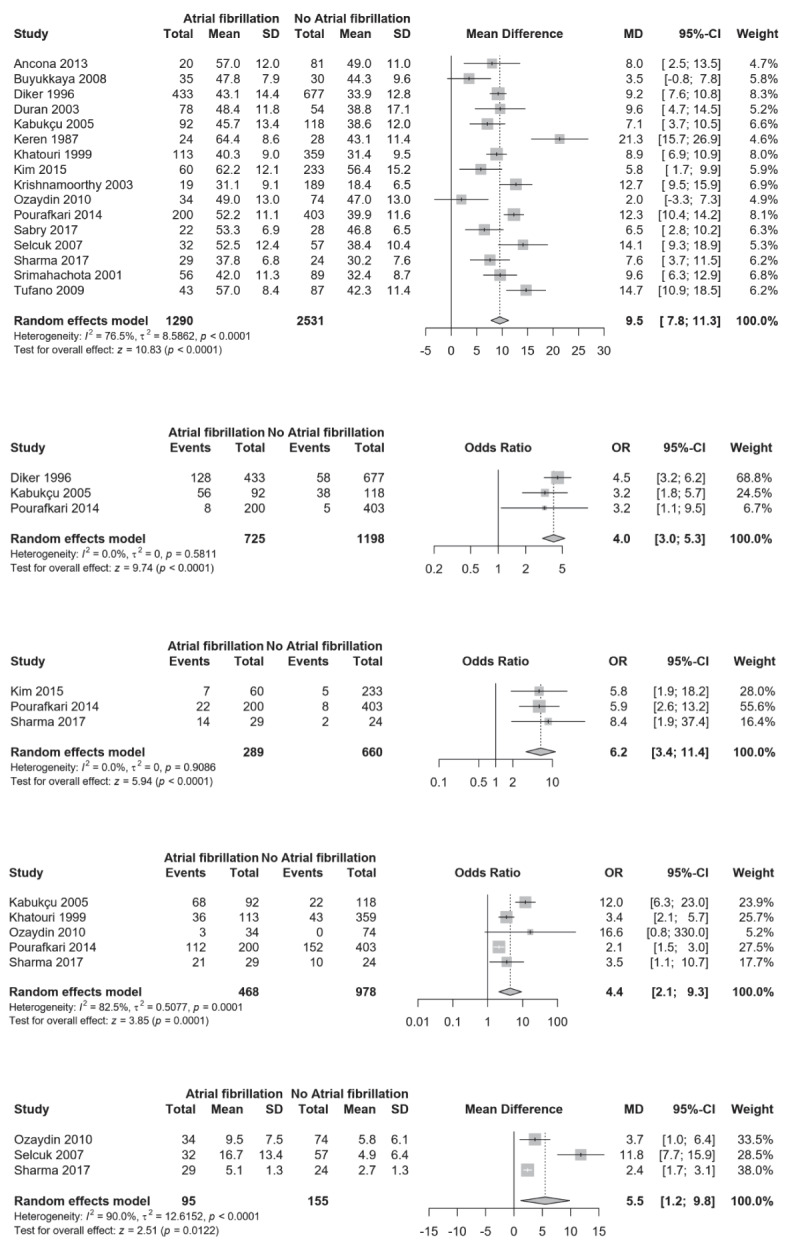
**Panel A.** Comparison of mean age between RHD patients with and without AF; **Panel B.** Comparison of tricuspid valve involvement between RHD patients with and without AF; **Panel C.** Comparison of previous cerebrovascular event* between RHD patients with and without AF; **Panel D.** Comparison of advanced heart failure** between RHD patients with and without AF; **Panel E.** Highly sensitive C reactive protein concentration (mg/dL).

**Figure 4:**
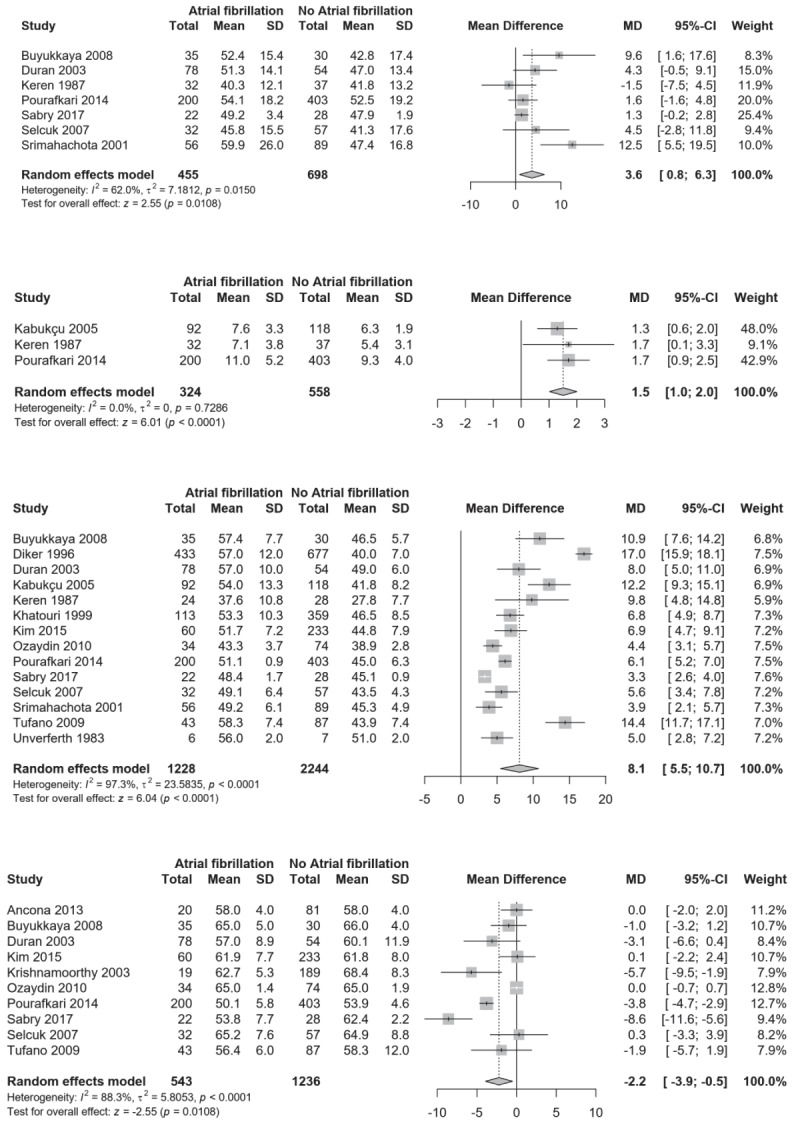
**Panel A.** Comparison of systolic pulmonary artery pressure between RHD patients with and without AF; **Panel B.** Comparison of right atrium pressure between RHD patients with and without AF; **Panel C.** Comparison of left atrium diameter between RHD patients with and without AF; **Panel D.** Comparison of left ventricle ejection fraction between RHD patients with and without AF.

In this analysis including 18 studies, we included 1,334 with AF and 2,591 without AF (controls). Patients with AF had higher age compared to those without (MD: 9.5 years; 95% CI: 7.8–11.3; I^2^: 76.5%) (Figure 3), while there was no sex difference (Supplementary Figure 5). Having AF was associated with more advanced stages of heart failure (New York Heart Association classification stage 3 or 4) (OR: 4.4; 95% CI 2.1–9.3; I^2^: 82.5%) (Figure 3), higher tricuspid valve involvement (OR: 4.0; 95% CI: 3.0–5.3; I^2^: 0.0%) and history of previous stroke or transient ischemic attack (OR: 6.2; 95% CI: 3.4–11.4; I^2^: 0.0%) (Figure 3). Patients with AF had higher concentration of highly sensitive C-reactive protein (MD: 5.5 mg/dL; 95% CI: 1.2–9.8; I^2^: 90.0%) (Figure 3), of N-terminal pro b-type natriuretic peptide (MD: 45.0 pg/mL; 95% CI: 28.8–61.2) (Supplementary Figure 6) and of interleukin-6 (p = 0.05) [94] than those without AF. Systolic pulmonary arterial pressure was higher in patients with AF (MD: 3.6 mmHg; 95% CI: 0.8–6.3; I^2^: 62.0%) (Figure 4) while there was no difference for diastolic pulmonary arterial pressure (Supplementary Figure 7) and for both left ventricle end-systolic and end-diastolic diameters (Supplementary Figures 8 and 9). Patients with AF had higher right atrium pressure (MD: 1.5 mmHg; 95% CI: 1.0–2.0; I^2^: 0.0%) and higher left atrium diameter (MD: 8.1 mm; 95% CI: 5.5–10.7; I^2^: 97.3%) (Figure 4); whereas they had lower mitral valve area (MD: -0.1; 95% CI: -0.2–0.0; I^2^: 88.3%) (Supplementary Figure 10) and left ventricle ejection fraction (MD: -2.2; 95% CI: -3.9–-0.5; I^2^: 88.3%) (Figure 4) compared to those without AF.

Only one study reported multivariate data on potential correlates of AF in patients with RHD. The independent correlates of AF included age (odds ratio [OR] = 1.14, 95% CI 1.05–1.25, p = 0.002), left ventricle systolic ejection fraction (OR = 0.92, 95% CI 0.87–0.97, p = 0.003), left atrium strain (OR = 7.53, 95% CI 4.47–12.69 p < 0.001), and right atrial pressure (OR = 1.09, 95% CI 1.02–1.17, p = 0.01) [10].

## Discussion

AF is a major complication of RHD, and a predictor of adverse outcomes such as stroke, peripheral embolism, heart failure and death [72]. This review shows that AF is very prevalent in RHD patients, affecting about one-third of them. Its incidence increases over time from ~5% at one year following diagnosis of RHD, to ~13% and ~31% at 5 years and 10 years, respectively. This prevalence of AF appears to be higher in RHD than in some other structural heart diseases such as cardiomyopathies. For instance, in a recent review, we estimated that AF affects 20%–25% of patients with dilated, hypertrophic and ischemic cardiomyopathies. In sub-Saharan Africa for instance, rheumatic heart disease is one of the leading causes of AF, reported in 20%–35% of cases in most series [102].

The prevalence of AF in RHD increases with the country’s development index; it is lowest in Africa and South-East Asia, where RHD is endemic. This is a striking finding which needs further investigation. Indeed, based on the results of studies done in the general population, there is a belief that AF is less frequent in sub-Saharan Africa compared to other regions [103]. However, this prevalence is probably underestimated as many cases of paroxysmal AF are missed by a single 12-lead electrocardiography, in these resource-restrained settings where Holter electrocardiography is only available in few tertiary care facilities [104]. In addition, in the absence of universal health coverage in most of these countries, 12-lead electrocardiography cannot always be repeated when necessary due to unaffordability. In this perspective, this presumed low detection rate of AF in RHD patients in developing countries is concerning, because undiagnosed AF is a missed opportunity for treatment and prevention of complications. However, further studies are needed to explore the reasons explaining the association between country human development index, geographic localization and the prevalence of AF in RHD patients as shown in this study.

This review shows that age is the most consistent correlates of AF across studies. We found that the prevalence of AF was higher in adults compared to children, and that the mean age of RHD patients with AF was higher than that of patients in sinus rhythm. Ageing is known as the strongest risk factor for AF in the general population, with the prevalence of AF increasing from 2% to 5% and 10% in people aged ≥ 40 years, ≥ 65 years, and ≥ 80 years, respectively [105]. Specifically, the association of increased prevalence of AF with age in RHD patients can be attributed to age-related increment in the severity of valvular lesions and a longer duration of inflammatory processes leading to increased chambers remodeling and eventually higher rates of AF. This is coupled with the AF risk inherent to ageing per se and to other co-morbidities. Indeed, although only demonstrated in one study, classic risk factors for AF such as diabetes mellitus, hypertension, and high BMI were significantly associated with AF in patients with rheumatic mitral stenosis [92].

Chronic inflammation is central in the pathogenesis of rheumatic heart disease. During acute rheumatic fever, an autoimmune response to untreated group A streptococcal pharyngitis causes inflammation and subsequently fibrosis of the heart valves. An association between inflammation and AF has been established [106]. The inflammation in the context of rheumatic carditis is therefore thought to be directly involved in the occurrence of AF in RHD [9,93,97]. This is suggested by inflammatory changes in rheumatic valve tissue and elevated plasma levels of CRP and circulating adhesion molecules in patients with rheumatic mitral stenosis [107,108,109]. AF might result from the myocardial disarrays caused by inflammatory and fibrotic changes in the atrial conduction system [90]. Few inflammatory markers including IL-6 and Hs-CRP correlate well with the presence of AF [9,94,98], and therefore have a potential clinical utility in AF prediction in patients with RHD, especially paroxysmal AF. NT-proBNP, a surrogate of left ventricular end-diastolic pressure, has also been identified as a possible predictor of AF but with a much lower correlation [31].

Left atrium enlargement (LAE) was associated with AF in our unadjusted data meta-analysis, and was an established independent predictor in multivariate analysis in several individual studies. In their study, Kim et al. found that left atrial dimension ≥ 47 mm predicted new-onset AF, as well as the composite of incident AF, systemic embolism and all-cause mortality in patients with rheumatic mitral stenosis in sinus rhythm [8]. Furthermore, a study suggested that LAE is associated with anticoagulation failure in AF patients with ischemic stroke [110]. All these data support the practice of anticoagulation in patients with rheumatic mitral stenosis in sinus rhythm but with left atrial diameter ≥ 50 mm or left atrial thrombus [86]. AF was more frequent in severe RHD disease, and consequently more in patients who had a valvular intervention as those patients had severe disease. Moreover, the prevalence of AF was much higher in mitral valve disease compared to aortic valve disease, because mitral valve disease, especially stenosis, directly causes LAE and subsequently AF.

The association of AF with advanced heart failure as indicated by NYHA III/IV functional classes is in keeping with the fact that AF is in one hand a factor of progression or decompensation to heart failure, and in the other hand a complication of heart failure in general and specifically in RHD [111]. Furthermore, for most hemodynamic parameters there was no significant difference between patients with AF and those in sinus rhythm. This is consistent with the idea that the poorer effort tolerance in patients with AF might be mainly due to an increase in ventricular rate and left atrial pressure during exercise [88].

The high incidence of AF in patients with RHD, and mainly those with rheumatic mitral stenosis, highlights the need for guidelines on the timing of clinical and electrocardiographic screening of AF in this population. Furthermore, because the duration of AF reduces the chances of sustained cardioversion success [112], a predictive tool to identify patients at high risk of developing AF might help in early detection and treatment. The likelihood of developing AF might also be considered in decision-making regarding the appropriate time of valvular intervention in patients with RHD [10,92,93]. Indeed, a recent study in patients with degenerative mitral regurgitation indicated that detection of AF, even paroxysmal, should trigger prompt consideration for surgery [113].

The high prevalence of AF in RHD patients emphasizes the high need for oral anticoagulation in this population. Oral anticoagulants have proven efficacy in preventing systemic stroke and peripheral embolism. Unfortunately, the uptake of anticoagulation by eligible RHD patients is suboptimal. For instance, the proportion of eligible patients receiving oral anticoagulants was 70% in the REMEDY study [86] and 77.8% in the HP-RHD registry [72]. Furthermore, according to the REMEDY study baseline data, only 37% of RHD patients with AF and on warfarin had 1–3 INR tests done in the last 6 months and 22.2% had a therapeutic INR (2.0–3.0 range) at enrollment [86]. However, the utilization of oral anticoagulant is higher in rheumatic valvular AF compared to non-valvular AF as reported in the RELY-AF registry where 58% of eligible patients were on warfarin [114].

This study has few limitations. There was marked heterogeneity across studies; we found that this heterogeneity was mainly explained by countries’ level of development, distribution across WHO regions, age groups, and RHD endemic profile of the countries. There was likely an underestimation of paroxysmal AF as most of the studies used a single 12-lead ECG recording. Furthermore, the data to estimate the incidence of AF in patients with RHD only came from few studies, limiting the accuracy of these estimates. Finally, our meta-analysis of the correlates of AF in RHD included unadjusted data. Therefore, confounders were not excluded, except in one study in which regression analysis was performed. Nevertheless, this study is the first to provide a comprehensive summary and estimation of the prevalence and incidence of AF in the patients with RHD, and to explore its correlates using strong statistical methods.

## Conclusion

This study shows that about one-third of patients with RHD have AF, with an incidence which almost triple every five years after diagnosis. The main correlates of AF include age, mitral valve disease, especially in the presence of associated tricuspid involvement, left atrium enlargement, right atrium pressure, and systolic pulmonary arterial pressure. AF is also associated with advanced heart failure and thromboembolism in patients with RHD. These findings call for guidelines on the timing of clinical and electrocardiographic screening of AF in this population, and for improved utilization of oral anticoagulation in those with AF. Furthermore, a predictive tool to identify patients at high risk of developing AF might help for early detection and treatment, as well as for decision-making regarding timing for valvular intervention.

## Data Accessibility Statemnt

All data generated or analyzed during this study are included in this published article and its supplementary information files.

## Additional Files

The additional files for this article can be found as follows:

10.5334/gh.807.s1 Supplementary Table 1. Search strategy in EMBASE.

10.5334/gh.807.s2 Supplementary Table 2. Summarized study characteristics.

10.5334/gh.807.s3 Supplementary Table 3. Individual characteristics of included studies.

10.5334/gh.807.s4 Supplementary Table 4. MOOSE checklist.

10.5334/gh.807.s5 Supplementary Figure 1. Studies selection.

10.5334/gh.807.s6 Supplementary Figure 2. Leave-one-out sensitivity analysis of the global prevalence of atrial fibrillation in rheumatic heart disease.

10.5334/gh.807.s7 Supplementary Figure 3. Funnel plot for publication bias.

10.5334/gh.807.s8 Supplementary Figure 4. Meta-analysis of the prevalence of atrial fibrillation in patients in rheumatic heart disease who had valvular interventions.

10.5334/gh.807.s9 Supplementary Figure 5. Comparison of proportion of female sex between patients with and without atrial fibrillation in RHD.

10.5334/gh.807.s10 Supplementary Figure 6. Comparison N-terminal pro b-type natriuretic peptide concentration between patients with and without atrial fibrillation in RHD.

10.5334/gh.807.s11 Supplementary Figure 7. Comparison of mean diastolic pulmonary arterial pressure (mmHg) between patients with and without atrial fibrillation in RHD.

10.5334/gh.807.s12 Supplementary Figure 8. Comparison of left ventricle end-systolic diameter between patients with and without atrial fibrillation in RHD.

10.5334/gh.807.s13 Supplementary Figure 9. Comparison of left ventricle end-diastolic diameter between patients with and without atrial fibrillation in RHD.

10.5334/gh.807.s14 Supplementary Figure 10. Comparison of mitral valve area (cm^2^) between patients with and without atrial fibrillation in RHD.
