# Supplementary material for: Meta-Analysis of the Incidence, Prevalence, and Correlates of Atrial Fibrillation in Rheumatic Heart Disease
Source: Glob Heart. 2020 May 18;15(1):38. doi: 10.5334/gh.807 (PMC7427678; doi:10.5334/gh.807)
Supplement: Supplementary Table 1. — Search strategy in EMBASE. [file gh-15-1-807-s1.pdf]

Meta-analysis of the incidence, prevalence and predictors of atrial fibrillation in rheumatic heart disease

Jean Jacques Noubiap, Ulrich Flore Nyaga, Aude Laetitia Ndoadoumgue,

Jan René Nkeck, Anderson Ngouo, Jean Joel Bigna

APPENDIX

Supplementary Tables

Supplementary Table 1. Search strategy in EMBASE 2
Supplementary Table 2. Summarized study characteristics 2
Supplementary Table 3. Individual characteristics of included studies 3
Supplementary Table 4. MOOSE checklist 7

Supplementary Figures

Supplementary Figure 1. Studies selection 9

Supplementary Figure 2. Leave-one-out sensitivity analysis of the global prevalence of atrial fibrillation in rheumatic heart disease 10

Supplementary Figure 4. Meta-analysis of the prevalence of atrial fibrillation in patients in rheumatic heart disease who had valvular interventions 12

Supplementary Figure 5. Comparison of proportion of female sex between patients with and without atrial fibrillation in RHD 12

Supplementary Figure 6. Comparison N-terminal pro b-type natriuretic peptide concentration between patients with and without atrial fibrillation in RHD 13

Supplementary Figure 7. Comparison of mean diastolic pulmonary arterial pressure (mmHg) between patients with and without atrial fibrillation in RHD 13

Supplementary Figure 8. Comparison of left ventricle end-systolic diameter between patients with and without atrial fibrillation in RHD 13

Supplementary Figure 9. Comparison of left ventricle end-diastolic diameter between patients with and without atrial fibrillation in RHD 13

Supplementary Figure 10. Comparison of mitral valve area (cm²) between patients with and without atrial fibrillation in RHD 14

Supplementary Table 1. Search strategy in EMBASE

Search	Search terms
#1	'rheumatic heart disease'/exp OR 'rheumatic heart disease' OR 'rhd' OR 'rheumatic mitral' OR 'rheumatic aortic' OR 'rheumatic tricuspid' OR 'rheumatic pulmonary' OR 'bouillaud disease' OR 'bouillauds disease'
#2	'atrial fibrillation'/exp OR 'atrial fibrillation' OR 'auricular fibrillation'/exp OR 'auricular fibrillation'
#3	#1 AND #2
