# Supplementary material for: Meta-Analysis of the Incidence, Prevalence, and Correlates of Atrial Fibrillation in Rheumatic Heart Disease
Source: Glob Heart. 2020 May 18;15(1):38. doi: 10.5334/gh.807 (PMC7427678; doi:10.5334/gh.807)
Supplement: Supplementary Table 2. — Summarized study characteristics. [file gh-15-1-807-s2.pdf]

Supplementary Table 2. Summarized study characteristics

**Characteristics**	**N = 83**
Year of publication, range	1984-2019
Period of inclusion of participants, range	1965-2017
Male, range (N = 64)	0-63
Design	
- Cross sectional	42
- Prospective cohort	30
- Retrospective cohort	11
Timing of data collection	
- Prospective	53
- Retrospective	27
- Unclear	3
Sampling method	
- Consecutive	74
- Unclear	9
Number of study sites	
- One site	68
- Multisite	6
- Unclear	9
Surgical intervention	
- Before	57
- After	29
