# Supplementary material for: Meta-Analysis of the Incidence, Prevalence, and Correlates of Atrial Fibrillation in Rheumatic Heart Disease
Source: Glob Heart. 2020 May 18;15(1):38. doi: 10.5334/gh.807 (PMC7427678; doi:10.5334/gh.807)
Supplement: Supplementary Table 3. — Individual characteristics of included studies. [file gh-15-1-807-s3.pdf]

Supplementary Table 3. Individual characteristics of included studies

Author	Year	Design	Country	Timing	Selection	Sites	Period	Method of diagnosis of AF	Surgical intervention	Population	Male	Sample size	Score on risk of bias
**Abbas**	2016	Cross sectional	Pakistan	Prospective	Consecutive	One site	2011	12 lead ECG	Yes	Adults	70	93	7
**Acartürk**	1997	Cross sectional	Turkey	Prospective	Consecutive	Unclear	NR	12 lead ECG	No	Adults	18.5	168	4
**Adhikari**	2016	Cross sectional	Nepal	Retrospective	Consecutive	One site	2013-2015	12 lead ECG	Yes	Children, Adolescents	46.6	131	6
**Adhikari**	2016	Cross sectional	Nepal	Prospective	Consecutive	One site	2014	12 lead ECG	No	Adults	54.6	74	8
**Arora**	1994	Prospective cohort	India	Prospective	Consecutive	One site	NR	NR	No	Children, Adolescents, Adults	25.6	600	8
**Arora**	2002	Retrospective cohort	India	Retrospective	Not clear	One site	NR	NR	Yes	Children, Adolescents, Adults	50	4850	2
**Augestad**	1999	Retrospective cohort	Russia	Retrospective	Consecutive	One site	1965-1993	NR	Yes	Adolescents, Adults	NR	296	3
**Behra**	2018	Cross sectional	India	Prospective	Consecutive	One site	2015-2016	12 lead ECG	No	Adults	31.7	268	8
**Boonyasirinant**	2007	Cross sectional	Thailand	Prospective	Consecutive	One site	2002-2004	12 lead ECG	No	Adults	29.6	260	6
**Bouleti**	2014	Prospective cohort	France	Prospective	Consecutive	Unclear	1986-1995	NR	Yes	Adults	17.2	1024	3
**Boyarchuk**	2019	Cross sectional	Ukraine	Prospective	Not clear	One site	NR	12 lead ECG	No	Adults	37.1	35	4
**Chen**	1998	Cross sectional	China	Prospective	Consecutive	One site	1985-1991	NR	Yes	Adults	29.7	202	4
**Chockalingam**	2003	Cross sectional	India	Retrospective	Consecutive	One site	NR	NR	No	Children, Adolescents, Adults	NR	10000	2
**Choudhary**	2003	Prospective cohort	India	Prospective	Consecutive	Unclear	1990-2001	NR	Yes	Children, Adolescents, Adults	49.3	276	4
**Chu**	2001	Prospective cohort	China	Prospective	Consecutive	One site	NR	NR	No	Adults	26	119	8

**Cruz-Gonzales**	2011	Prospective cohort	Spain	Prospective	Consecutive	Unclear	1986-2005	NR	Yes	Adults	17	1015	5
**De Santos**	2004	Prospective cohort	Italy	Prospective	Consecutive	One site	1975-2003	12 lead ECG	Both	Adults	0	267	7
**Diker**	1996	Cross sectional	Turkey	Retrospective	Consecutive	One site	1990-1994	12 lead ECG	No	Adults	26.9	1110	6
**Doukky**	2014	Retrospective cohort	USA	Retrospective	Consecutive	One site	2005-2011	NR	No	Adults	14.4	90	5
**Drighil**	2012	Prospective cohort	Morocco	Prospective	Consecutive	One site	2009	NR	No	Adults	11.9	59	4
**Esteves**	2017	Prospective cohort	Brazil	Prospective	Consecutive	One site	2008-2014	NR	Yes	Adults	12	142	7
**Fawzy**	2009	Prospective cohort	Saudi Arabia	Prospective	Consecutive	One site	1989-2005	NR	No	Adults	25	474	5
**Gamra**	2003	Prospective cohort	Tunisia	Prospective	Not clear	One site	1987-1999	NR	No	Adults	27	544	4
**Goswami**	2004	Cross sectional	India	Prospective	Consecutive	Unclear	NR	NR	No	Adults		200	1
**Goswani**	2000	Cross sectional	India	Prospective	Consecutive	One site	NR	NR	No	Children, Adolescents, Adults	56	200	8
**Guteta**	2016	Retrospective cohort	Ethiopia	Retrospective	Not clear	One site	1983-2013	NR	Yes	Adults	35.6	105	1
**Hernandez**	1993	Cross sectional	Spain	Not reported/Unclear	Consecutive	One site	NR	NR	Yes	Adults	21	335	2
**Hung**	1991	Prospective cohort	Taiwan	Prospective	Not clear	One site	1987-1989	NR	No	Adults	26.9	219	3
**Islam**	2010	Prospective cohort	Bangladesh	Prospective	Consecutive	One site	2002-2003	12 lead ECG	No	Children, Adolescents, Adults	48	50	6
**Iung**	2004	Retrospective cohort	France	Retrospective	Consecutive	One site	1986-2001	NR	No	Adults	19.8	2773	5
**Kafle**	2016	Cross sectional	Nepal	Retrospective	Consecutive	One site	2009-2015	NR	No	Children, Adolescents, Adults	26.27	454	4

**Kim**	2018	Retrospective cohort	South Korea	Retrospective	Consecutive	One site	1980-2013	NR	Yes	Adults	25	742	3
**Lim**	2001	Prospective cohort	UK	Prospective	Consecutive	One site	1987-1999	12 lead ECG	Yes	Adults	62.5	26	7
**Lin**	1992	Cross sectional	Taiwan	Prospective	Consecutive	One site	NR	NR	Yes	Adults	35.45	110	6
**López-meneses**	2009	Prospective cohort	Mexico	Prospective	Consecutive	One site	1993-2005	NR	No	Adults	88	61	4
**Luo**	2017	Prospective cohort	China	Prospective	Consecutive	One site	2012-2016	NR	Yes	Adults	25.7	179	6
**Mahmoud Elsayed**	2017	Cross sectional	Egypt	Prospective	Not clear	One site	2016-2017	NR	Yes	Adults	2	30	3
**Malik**	2005	Prospective cohort	Pakistan	Prospective	Consecutive	One site	2003	NR	Yes	Children, Adolescents, Adults	23.68	76	2
**Melka**	1996	Cross sectional	Ethiopia	Prospective	Consecutive	One site	1994-1995	NR	No	Children, Adolescents, Adults	34	114	8
**Mrozowska**	1999	Cross sectional	Poland	Not reported/Unclear	Consecutive	Unclear	NR	NR	No	Adults	NR	141	3
**Negi**	2018	Cross sectional	India	Retrospective	Consecutive	One site	2011-2016	NR	No	Children, Adolescents, Adults	27.7	2005	4
**Okello**	2013	Cross sectional	Uganda	Prospective	Consecutive	One site	2010-2012	12 lead ECG	No	Adults	36.6	309	5
**Okello**	2017	Prospective cohort	Uganda	Prospective	Not clear	One site	2011-2013	12 lead ECG	No	Children, Adolescents, Adults	33.2	331	4
**Okubo**	1984	Cross sectional	Bangladesh	Retrospective	Consecutive	One site	NR	NR	No	Children, Adolescents, Adults	38.53	205	3
**Ostovan**	2014	Prospective cohort	Iran	Prospective	Consecutive	One site	2011-2012	NR	Yes	Adults	18	50	6
**Ozaydin**	2010	Cross sectional	Turkey	Prospective	Consecutive	One site	2008	12 lead ECG	No	Adults	29.8	108	8
**Ozkan**	1998	Prospective cohort	Turkey	Prospective	Consecutive	One site	1995-1997	NR	Both	Adults	34.9	169	4
**Pourafkari**	2015	Cross sectional	Iran	Retrospective	Consecutive	One site	2002-2012	12 lead ECG	No	Adults	22.8	603	5

**Pourafkari**	2018	Retrospective cohort	Iran	Retrospective	Consecutive	One site	2005-2017	12 lead ECG	No	Adolescents, Adults	24	1312	7
**Rajbhandari**	2006	Cross sectional	Nepal	Not reported/Unclear	Consecutive	Unclear	2003-2004	NR	Yes	Children, Adolescents, Adults	26	200	2
**Ranganayakulu**	2016	Prospective cohort	India	Prospective	Not clear	One site	2012-2013	NR	Yes	Adolescents, Adults	19	100	4
**Russell**	2015	Retrospective cohort	Australia	Retrospective	Consecutive	Multisite	2001-2012	NR	Yes	Adults	35.5	1384	2
**Russell**	2017	Retrospective cohort	Australia	Retrospective	Consecutive	Multisite	2001-2013	NR	Yes	Children, Adolescents, Adults	NR	1594	3
**Sancho**	1990	Prospective cohort	Argentina	Prospective	Consecutive	One site	NR	NR	Both	Adults	34	197	8
**Sarralde**	2010	Retrospective cohort	Spain	Retrospective	Consecutive	One site	1974-2007	NR	Yes	Adults	16.4	299	4
**Shang**	2005	Prospective cohort	China	Prospective	Consecutive	Unclear	NR	NR	Yes	Adults	NR	30	1
**Sharma**	2015	Cross sectional	India	Retrospective	Consecutive	One site	1999-2005	12 lead ECG	Yes	Children, Adolescents, Adults	41.5	2330	3
**Sharma**	2015	Cross sectional	India	Prospective	Consecutive	One site	NR	12 lead ECG	No	Adolescents, Adults	39.33	94	6
**Shimada**	1986	Prospective cohort	Japan	Prospective	Consecutive	Multisite	1971-1984	12 lead ECG	No	Adults	38.5	301	5
**Sims**	2006	Cross sectional	USA	Retrospective	Consecutive	One site	1993-2005	12 lead ECG	No	Adults	12.5	104	5
**Sliwa**	2010	Prospective cohort	South Africa	Prospective	Consecutive	One site	2006-2007	12 lead ECG	No	Adults	32	344	4
**Souza**	2011	Cross sectional	Brazil	Retrospective	Consecutive	One site	1990-1994	12 lead ECG	Yes	Adolescents, Adults	18	50	3
**Srimahachota**	2001	Cross sectional	Thailand	Retrospective	Consecutive	One site	1993-1997	NR	Yes	Adults		145	3
**Tadele**	2013	Cross sectional	Ethiopia	Retrospective	Consecutive	One site	2009-2012	12 lead ECG	No	Children, Adolescents	41.9	365	3
**Tomai**	2014	Prospective	Italy	Prospective	Consecutive	One site	1991-2011	NR	Yes	Adolescents, Adults	17.4	527	7

		cohort											
**Vijayvergiya**	2011	Prospective cohort	India	Prospective	Consecutive	One site	2006-2007	NR	No	Adolescents, Adults	44.6	47	7
**Yadeta**	2019	Cross sectional	Ethiopia	Retrospective	Consecutive	One site	2016	12 lead ECG	No	Children, Adolescents, Adults	28	500	7
**Zhang**	2013	Cross sectional	Uganda	Prospective	Consecutive	One site	2011-2012	12 lead ECG	No	Adults	27.7	130	7
**Zhou**	2008	Prospective cohort	China	Prospective	Consecutive	Multisite	NR	12 lead ECG	No	Adults		29079	8
**Zuhlke**	2016	Prospective cohort	Egypt; Ethiopia; Kenya; India; Malawi; Mozambique; Namibia; Nigeria; Rwanda; Uganda; South Africa; Sudan; Yemen; Zambia;	Prospective	Consecutive	Multisite	2010-2012	NR	No	Adults	33.3	3343	6

NR: not reported; AF: atrial fibrillation; ECG: electrocardiogram
