# Supplementary material for: Meta-Analysis of the Incidence, Prevalence, and Correlates of Atrial Fibrillation in Rheumatic Heart Disease
Source: Glob Heart. 2020 May 18;15(1):38. doi: 10.5334/gh.807 (PMC7427678; doi:10.5334/gh.807)
Supplement: Supplementary Table 4. — MOOSE checklist. [file gh-15-1-807-s4.pdf]

Supplementary Table 4. MOOSE checklist

Item No	Recommendation	Reported on Page No
Reporting of background should include		
1	Problem definition	3
2	Hypothesis statement	3
3	Description of study outcome(s)	3
4	Type of exposure or intervention used	3
5	Type of study designs used	3
6	Study population	3
Reporting of search strategy should include		
7	Qualifications of searchers (eg, librarians and investigators)	14
8	Search strategy, including time period included in the synthesis and key words	4
9	Effort to include all available studies, including contact with authors	4
10	Databases and registries searched	4
11	Search software used, name and version, including special features used (eg, explosion)	4
12	Use of hand searching (eg, reference lists of obtained articles)	4
13	List of citations located and those excluded, including justification	7
14	Method of addressing articles published in languages other than English	4
15	Method of handling abstracts and unpublished studies	4
16	Description of any contact with authors	4
Reporting of methods should include		
17	Description of relevance or appropriateness of studies assembled for assessing the hypothesis to be tested	5
18	Rationale for the selection and coding of data (eg, sound clinical principles or convenience)	5
19	Documentation of how data were classified and coded (eg, multiple raters, blinding and interrater reliability)	5
20	Assessment of confounding (eg, comparability of cases and controls in studies where appropriate)	5
21	Assessment of study quality, including blinding of quality assessors, stratification or regression on possible predictors of study results	5
22	Assessment of heterogeneity	6
23	Description of statistical methods (eg, complete description of fixed or random effects models, justification of whether the chosen models account for predictors of study results, dose-response models, or cumulative meta-analysis) in sufficient detail to be replicated	6
24	Provision of appropriate tables and graphics	6
Reporting of results should include		
25	Graphic summarizing individual study estimates and overall estimate	7-10
26	Table giving descriptive information for each study included	7-10
27	Results of sensitivity testing (eg, subgroup analysis)	7-9
28	Indication of statistical uncertainty of findings	7-10

Item No	Recommendation	Reported on Page No
Reporting of discussion should include		
29	Quantitative assessment of bias (eg, publication bias)	7
30	Justification for exclusion (eg, exclusion of non-English language citations)	7
31	Assessment of quality of included studies	7
Reporting of conclusions should include		
32	Consideration of alternative explanations for observed results	10-13
33	Generalization of the conclusions (ie, appropriate for the data presented and within the domain of the literature review)	14
34	Guidelines for future research	10-14
35	Disclosure of funding source	14
