# Supplementary figures and images for: Meta-Analysis of the Incidence, Prevalence, and Correlates of Atrial Fibrillation in Rheumatic Heart Disease
Source: Glob Heart. 2020 May 18;15(1):38. doi: 10.5334/gh.807 (PMC7427678; doi:10.5334/gh.807)

**Supplementary Figure 1. Studies selection**

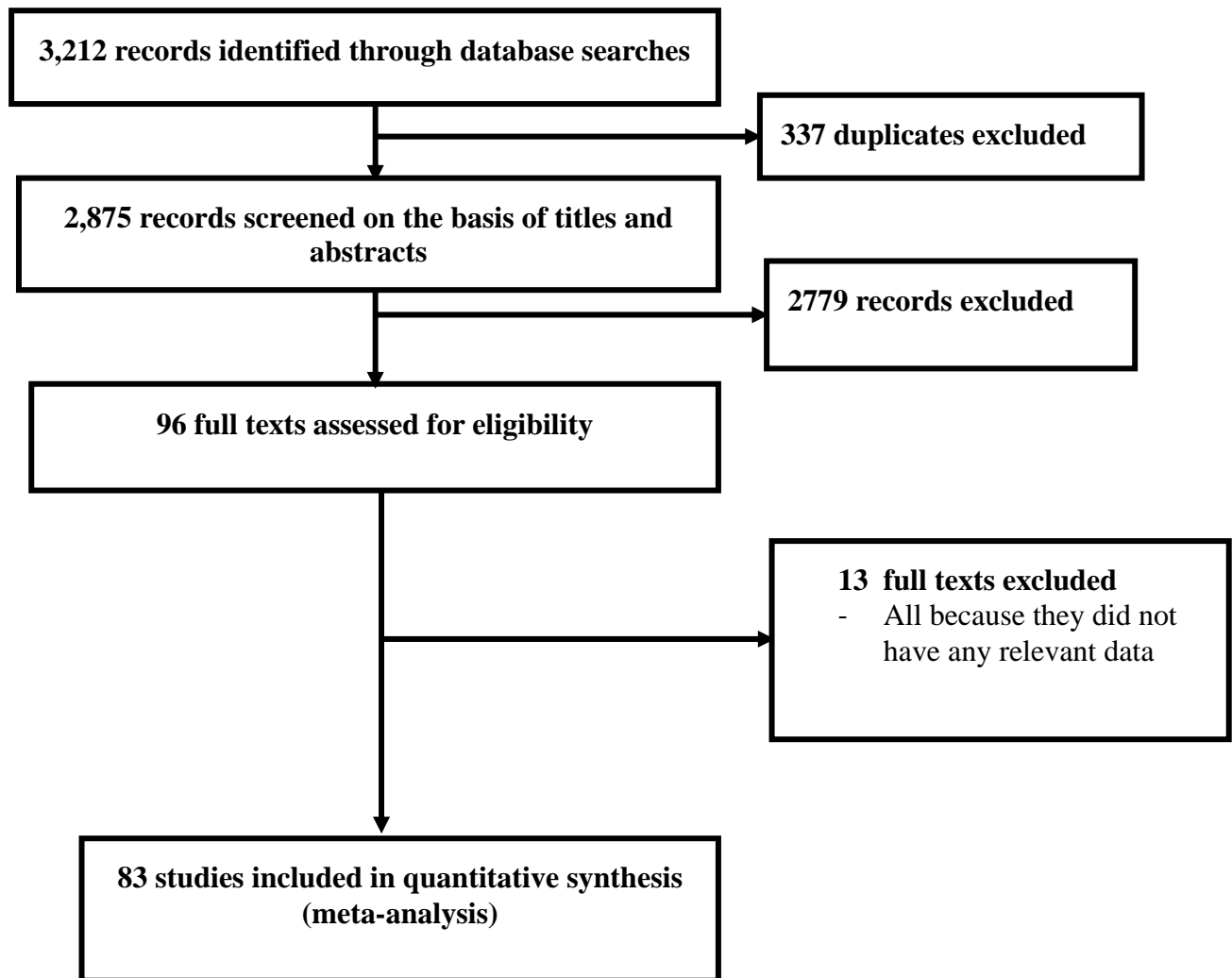

Supplement: Supplementary Figure 1. — Studies selection. [file gh-15-1-807-s5.pdf]

Supplementary Figure 3. Funnel plot for publication bias

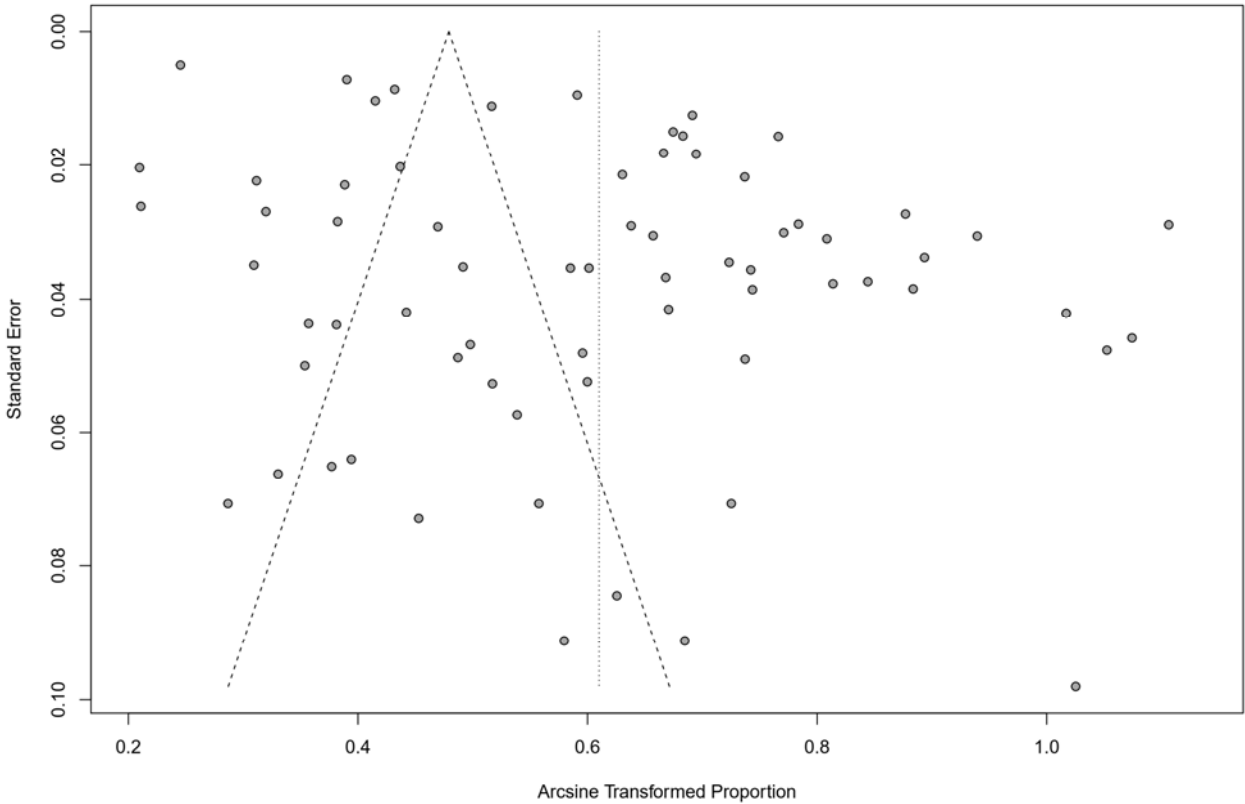

Supplement: Supplementary Figure 3. — Funnel plot for publication bias. [file gh-15-1-807-s7.pdf]
