# Supplementary material for: Meta-Analysis of the Incidence, Prevalence, and Correlates of Atrial Fibrillation in Rheumatic Heart Disease
Source: Glob Heart. 2020 May 18;15(1):38. doi: 10.5334/gh.807 (PMC7427678; doi:10.5334/gh.807)

Supplementary Figure 2. Leave-one-out sensitivity analysis of the global prevalence of atrial fibrillation in rheumatic heart disease

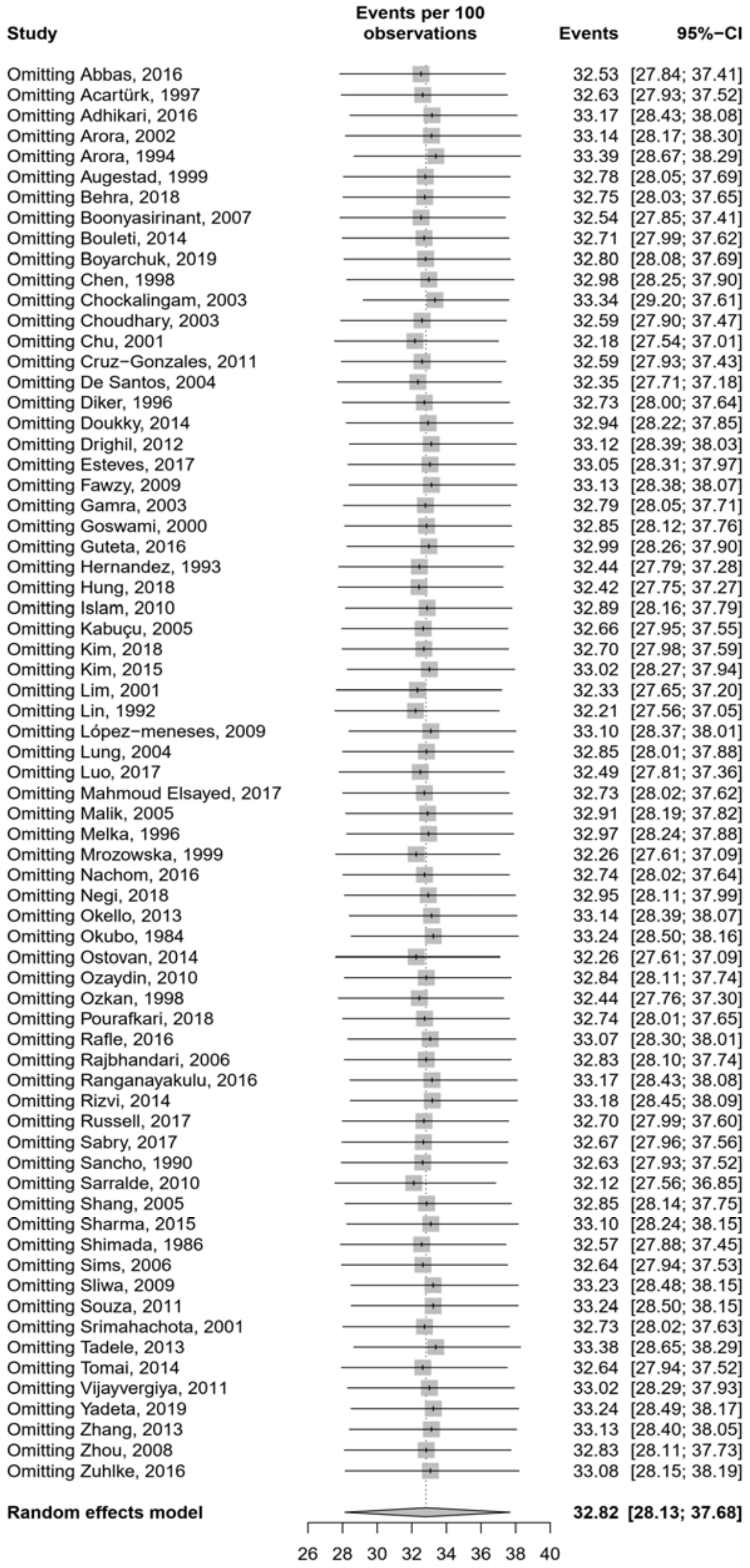

Supplement: Supplementary Figure 2. — Leave-one-out sensitivity analysis of the global prevalence of atrial fibrillation in rheumatic heart disease. [file gh-15-1-807-s6.pdf]
