# Supplementary material for: Meta-Analysis of the Incidence, Prevalence, and Correlates of Atrial Fibrillation in Rheumatic Heart Disease
Source: Glob Heart. 2020 May 18;15(1):38. doi: 10.5334/gh.807 (PMC7427678; doi:10.5334/gh.807)

Supplementary Figure 5. Comparison of proportion of female sex between patients with and without atrial fibrillation in RHD

Study	Atrial fibrillation Events	Atrial fibrillation Total	No Atrial fibrillation Events	No Atrial fibrillation Total	OR	95%-CI	Weight
Buyukkaya 2008	26	35	23	30	0.9	[0.3; 2.7]	5.0%
Duran 2003	55	78	25	54	2.7	[1.3; 5.6]	10.4%
Kabukçu 2005	68	92	88	118	1.0	[0.5; 1.8]	12.9%
Kim 2015	44	60	172	233	1.0	[0.5; 1.8]	12.4%
Krishnamoorthy 2003	14	19	128	189	1.3	[0.5; 3.5]	5.7%
Ozaydin 2010	22	34	56	74	0.6	[0.2; 1.4]	7.6%
Pourafkari 2014	148	200	317	403	0.8	[0.5; 1.1]	22.1%
Sabry 2017	15	22	17	28	1.4	[0.4; 4.3]	4.7%
Selcuk 2007	25	32	49	57	0.6	[0.2; 1.7]	5.2%
Sims 2006	34	38	45	53	1.4	[0.4; 4.9]	4.2%
Tufano 2009	25	43	56	87	0.8	[0.4; 1.6]	9.8%
**Random effects model**		**653**		**1326**	**1.0**	**[0.8; 1.3]**	**100.0%**

Heterogeneity: $I^2 = 21.8\%$, $\tau^2 = 0.0415$, $p = 0.2361$

Test for overall effect: $z = -0.15$ ($p = 0.8810$)

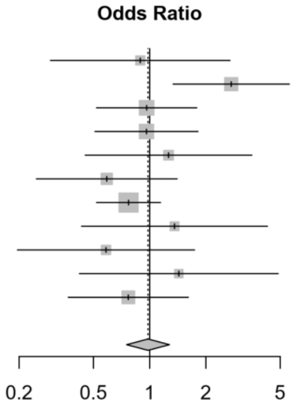

Supplement: Supplementary Figure 5. — Comparison of proportion of female sex between patients with and without atrial fibrillation in RHD. [file gh-15-1-807-s9.pdf]
