# Supplementary material for: Meta-Analysis of the Incidence, Prevalence, and Correlates of Atrial Fibrillation in Rheumatic Heart Disease
Source: Glob Heart. 2020 May 18;15(1):38. doi: 10.5334/gh.807 (PMC7427678; doi:10.5334/gh.807)

Supplementary Figure 6. Comparison N-terminal pro b-type natriuretic peptide concentration between patients with and without atrial fibrillation in RHD

	Atrial fibrillation			No Atrial fibrillation					
Study	Total	Mean	SD	Total	Mean	SD	Mean Difference	MD	95%−CI
Ozaydin 2010	34	69.9	56.9	74	24.9	29.3		45.0	[28.8; 61.2]

Heterogeneity: I^2 = NA%, τ^2 = NA

Test for overall effect: $z = 5.43$ ($p < 0.0001$)

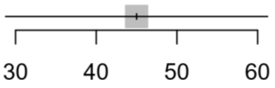

Supplement: Supplementary Figure 6. — Comparison N-terminal pro b-type natriuretic peptide concentration between patients with and without atrial fibrillation in RHD. [file gh-15-1-807-s10.pdf]
