# Supplementary material for: Meta-Analysis of the Incidence, Prevalence, and Correlates of Atrial Fibrillation in Rheumatic Heart Disease
Source: Glob Heart. 2020 May 18;15(1):38. doi: 10.5334/gh.807 (PMC7427678; doi:10.5334/gh.807)

Supplementary Figure 7. Comparison of mean diastolic pulmonary arterial pressure (mmHg) between patients with and without atrial fibrillation in RHD

Study	Atrial fibrillation Total	Atrial fibrillation Mean	Atrial fibrillation SD	No Atrial fibrillation Total	No Atrial fibrillation Mean	No Atrial fibrillation SD	MD	95%-CI	Weight
Keren 1987	32	20.3	6.5	37	19.6	7.0	0.7	[-2.5; 3.9]	29.6%
Pourafkari 2014	200	25.7	8.6	403	25.0	9.8	0.7	[-0.9; 2.3]	43.7%
Srimahachota 2001	56	28.1	12.8	89	22.7	9.2	5.4	[1.8; 9.0]	26.7%
**Random effects model**	**288**			**529**			**2.0**	**[-0.7; 4.6]**	**100.0%**

Heterogeneity: $I^2 = 64.9\%$, $\tau^2 = 3.5762$, $p = 0.0581$
Test for overall effect: $z = 1.44$ ($p = 0.1505$)

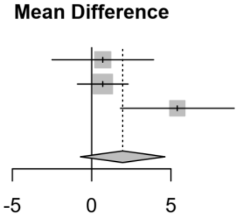

Supplement: Supplementary Figure 7. — Comparison of mean diastolic pulmonary arterial pressure (mmHg) between patients with and without atrial fibrillation in RHD. [file gh-15-1-807-s11.pdf]
