# Supplementary material for: Meta-Analysis of the Incidence, Prevalence, and Correlates of Atrial Fibrillation in Rheumatic Heart Disease
Source: Glob Heart. 2020 May 18;15(1):38. doi: 10.5334/gh.807 (PMC7427678; doi:10.5334/gh.807)

Supplementary Figure 8. Comparison of left ventricle end-systolic diameter between patients with and without atrial fibrillation in RHD

	Atrial fibrillation			No Atrial fibrillation					
**Study**	**Total**	**Mean**	**SD**	**Total**	**Mean**	**SD**	**MD**	**95%-CI**	**Weight**
Diker 1996	433	32.0	14.0	677	30.0	15.0	2.0	[0.2; 3.8]	16.9%
Kabukçu 2005	92	34.4	7.5	118	30.9	7.5	3.5	[1.5; 5.5]	15.9%
Kim 2015	60	31.2	6.1	233	31.4	5.1	-0.2	[-1.7; 1.3]	17.8%
Pourafkari 2014	200	30.3	9.3	403	31.5	7.9	-1.2	[-2.6; 0.2]	18.1%
Selcuk 2007	32	28.7	4.2	57	29.5	4.2	-0.8	[-2.6; 1.0]	16.7%
Tufano 2009	43	35.2	6.0	87	31.2	7.0	4.0	[1.6; 6.4]	14.5%
**Random effects model**	**860**			**1575**			**1.1**	**[-0.6; 2.8]**	**100.0%**

Heterogeneity: $I^2 = 81.5\%$, $\tau^2 = 3.5964$, $p < 0.0001$

Test for overall effect: $z = 1.26$ ($p = 0.2074$)

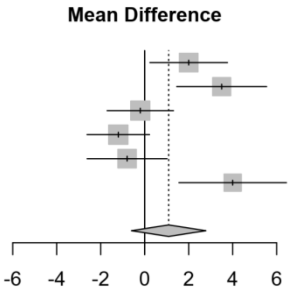

Supplement: Supplementary Figure 8. — Comparison of left ventricle end-systolic diameter between patients with and without atrial fibrillation in RHD. [file gh-15-1-807-s12.pdf]
