# Supplementary material for: Meta-Analysis of the Incidence, Prevalence, and Correlates of Atrial Fibrillation in Rheumatic Heart Disease
Source: Glob Heart. 2020 May 18;15(1):38. doi: 10.5334/gh.807 (PMC7427678; doi:10.5334/gh.807)

Supplementary Figure 9. Comparison of left ventricle end-diastolic diameter between patients with and without atrial fibrillation in RHD

Study	Atrial fibrillation Total	Mean	SD	No Atrial fibrillation Total	Mean	SD	MD	95%-CI	Weight
Ancona 2013	20	46.0	4.0	81	48.0	7.0	-2.0	[-5.2; 1.2]	10.1%
Diker 1996	433	51.0	21.0	677	49.0	19.0	2.0	[-0.4; 4.4]	13.1%
Kabukçu 2005	92	52.3	8.7	118	47.7	8.7	4.6	[2.2; 7.0]	13.1%
Kim 2015	60	50.5	6.0	233	49.8	5.8	0.7	[-1.0; 2.4]	16.2%
Pourafkari 2014	200	44.0	9.5	403	42.1	10.5	1.9	[0.2; 3.6]	15.9%
Selcuk 2007	32	44.7	3.8	57	46.0	4.4	-1.3	[-3.1; 0.5]	15.5%
Tufano 2009	43	48.8	3.9	87	47.5	5.0	1.3	[-0.4; 3.0]	16.0%
**Random effects model**	**880**			**1656**			**1.1**	**[-0.3; 2.5]**	**100.0%**

Heterogeneity: $I^2 = 70.5\%$, $\tau^2 = 2.4787$, $p = 0.0024$
Test for overall effect: $z = 1.51$ ($p = 0.1311$)

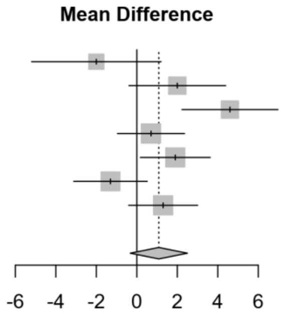

Supplement: Supplementary Figure 9. — Comparison of left ventricle end-diastolic diameter between patients with and without atrial fibrillation in RHD. [file gh-15-1-807-s13.pdf]
