# Supplementary material for: Meta-Analysis of the Incidence, Prevalence, and Correlates of Atrial Fibrillation in Rheumatic Heart Disease
Source: Glob Heart. 2020 May 18;15(1):38. doi: 10.5334/gh.807 (PMC7427678; doi:10.5334/gh.807)

**Supplementary Figure 10. Comparison of mitral valve area (cm²) between patients with and without atrial fibrillation in RHD**

| Study | Atrial fibrillation Total | Atrial fibrillation Mean | Atrial fibrillation SD | No Atrial fibrillation Total | No Atrial fibrillation Mean | No Atrial fibrillation SD | MD | 95%-CI | Weight |
|---|---|---|---|---|---|---|---|---|---|
| Ancona 2013 | 20 | 1.4 | 0.4 | 81 | 1.8 | 0.4 | -0.4 | [-0.6; -0.2] | 6.4% |
| Buyukkaya 2008 | 35 | 1.4 | 0.3 | 30 | 1.6 | 0.4 | -0.2 | [-0.3; 0.0] | 7.3% |
| Diker 1996 | 433 | 2.2 | 1.4 | 677 | 2.6 | 1.5 | -0.4 | [-0.6; -0.2] | 7.0% |
| Kabukçu 2005 | 92 | 1.3 | 0.4 | 118 | 1.4 | 0.4 | -0.1 | [-0.2; 0.0] | 8.9% |
| Keren 1987 | 24 | 1.6 | 0.3 | 28 | 1.6 | 0.4 | 0.0 | [-0.2; 0.2] | 6.6% |
| Kim 2015 | 60 | 1.4 | 0.4 | 233 | 1.5 | 0.5 | -0.1 | [-0.2; 0.0] | 8.1% |
| Krishnamoorthy 2003 | 19 | 0.7 | 0.2 | 189 | 0.7 | 0.2 | 0.0 | [-0.1; 0.1] | 9.3% |
| Ozaydin 2010 | 34 | 1.7 | 0.4 | 74 | 1.7 | 0.3 | -0.1 | [-0.2; 0.1] | 8.1% |
| Pourafkari 2014 | 200 | 0.9 | 0.2 | 403 | 0.9 | 0.2 | 0.0 | [ 0.0; 0.0] | 10.5% |
| Selcuk 2007 | 32 | 1.3 | 0.2 | 57 | 1.3 | 0.3 | -0.0 | [-0.2; 0.1] | 8.3% |
| Sharma 2017 | 29 | 0.7 | 0.2 | 24 | 0.8 | 0.2 | -0.1 | [-0.2; 0.0] | 9.2% |
| Tufano 2009 | 43 | 1.1 | 0.2 | 87 | 1.0 | 0.0 | 0.2 | [ 0.1; 0.2] | 10.3% |
| **Random effects model** | **1021** | | | **2001** | | | **-0.1** | **[-0.2; 0.0]** | **100.0%** |

Heterogeneity: $I^2 = 88.3\%$, $\tau^2 = 0.0151$, $p < 0.0001$

Test for overall effect: $z = -2.07$ ($p = 0.0383$)

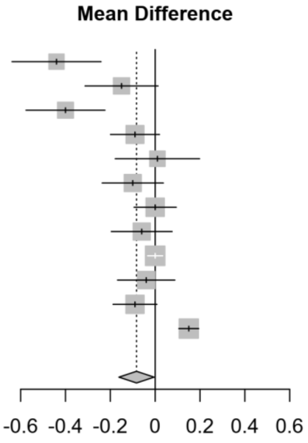

Supplement: Supplementary Figure 10. — Comparison of mitral valve area (cm2) between patients with and without atrial fibrillation in RHD. [file gh-15-1-807-s14.pdf]
